# Supplementary material for: Estrogens Counteract Platinum-Chemosensitivity by Modifying the Subcellular Localization of MDM4
Source: Cancers (Basel). 2019 Sep 12;11(9):1349. doi: 10.3390/cancers11091349 (PMC6770881; doi:10.3390/cancers11091349)
Supplement: Supplementary file 1 [file cancers-11-01349-s001.pdf]

SUPPLEMENTARY DATA

Estrogens counteract platinum-chemosensitivity by modifying subcellular localization of MDM4

Rossella Lucà, Giorgia di Blasio, Daniela Gallo, Valentina Monteleone, Isabella Manni, Laura Fici, Marianna Buttarelli, Germana Ciolli, Marsha Pellegrino, Emanuela Teveroni, Silvia Maiullari, Alessandra Ciucci, Alessandro Apollo, Francesca Mancini, Maria Pia Gentileschi, Gian Franco Zannoni, Alfredo Pontecorvi, Giovanni Scambia, Fabiola Moretti

This supplementary data contains:

Supplementary Figure S1

Supplementary Figure S2

Supplementary Figure S3

Supplementary Figure S4

Supplementary Figure S5

Supplementary Table S1

Supplementary Table S2

Supplementary Table S3

Supplementary Table S4

Uncropped gels of western blot Figures

Densitometric analyses

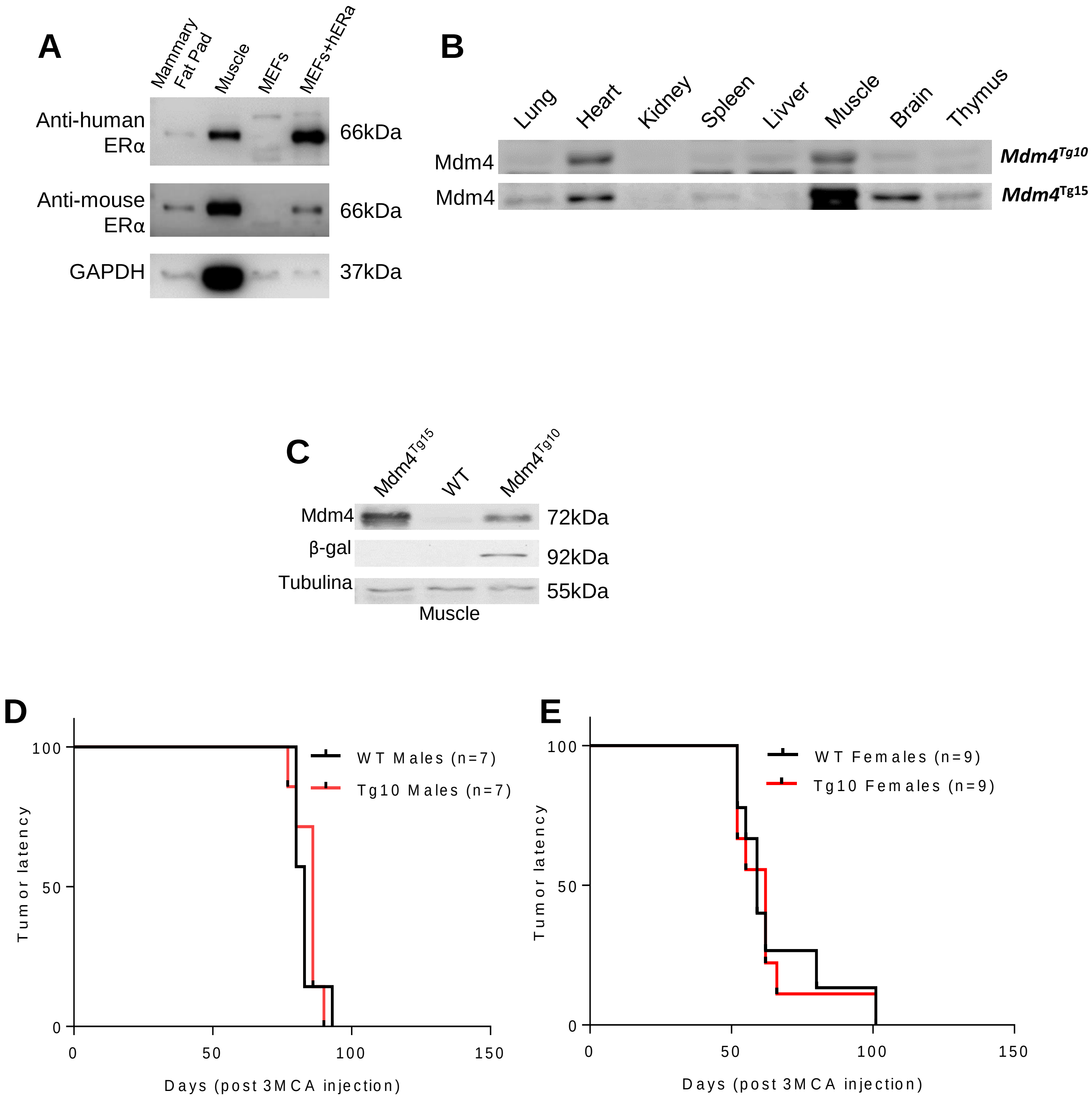

Figure S1. Analysis of protein and tumor latency in WT and $Mdm4^{Tg}$ mice**. A,** Levels of murine estrogen receptor alpha (ERα) in indicated tissue and cells. **B, C,** Western blot (WB) analysis of Mdm4 immunoprecipitated from indicated tissues (B) and in hindlimb muscle (C) from age-matched WT, $Mdm4^{Tg10}$ and $Mdm4^{Tg15}$ animals. β-gal signal is referred to the $Mdm4^{Tg10}$ mice that express the transgene flanked by LacZ cDNA. **D, E,** Tumor latency in wildtype (WT) and transgenic ($Mdm4^{Tg10}$) males (D) and females (E). Fibrosarcomas were induced by single injection of 3MCA in the hindlimb muscle of age-matched animals (Log-rank test, (D) df=1 χ^2= 0,3539 p=0,5519, (E) df=1 χ^2= 0,02072 p=0,8855). The experiments are representative of 3 (D) and 4 (E) independent experiments.

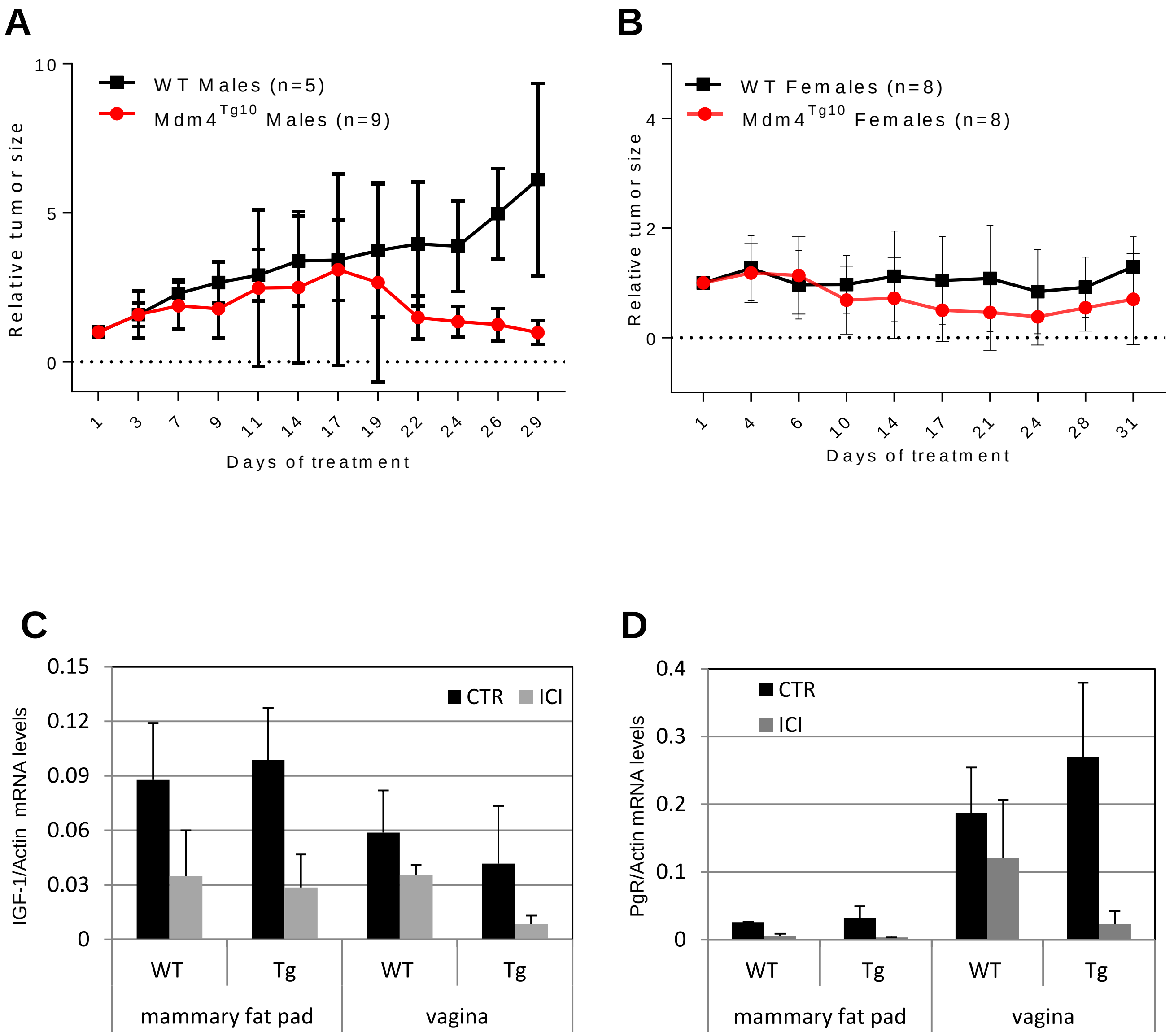

Figure S2. Growth curve of fibrosarcoma and mRNA levels of estrogen-targets in WT and Mdm4^{Tg} mice. **A,B,** Growth curve of fibrosarcoma in WT and Mdm4Tg^{10} males (A) and females (B) treated with cisplatin. After reaching a volume of approximately 200mm^3, animals were treated bi-weekly with i.p. cisplatin (5mg/Kg). Tumor size is relative to the volume at the first treatment (two-way ANOVA, (A) DF=11 F(interaction)=2,324 p= 0.0124; (B) DF=9 F(interaction)=0.6741 p= 0.734). **C, D**, qPCR of IGF-1 (C) and PgR (D) mRNA extracted from indicated tissue and organ of WT and Mdm4Tg^{15} females treated as indicated. Graphs show mean±SD (n=2).

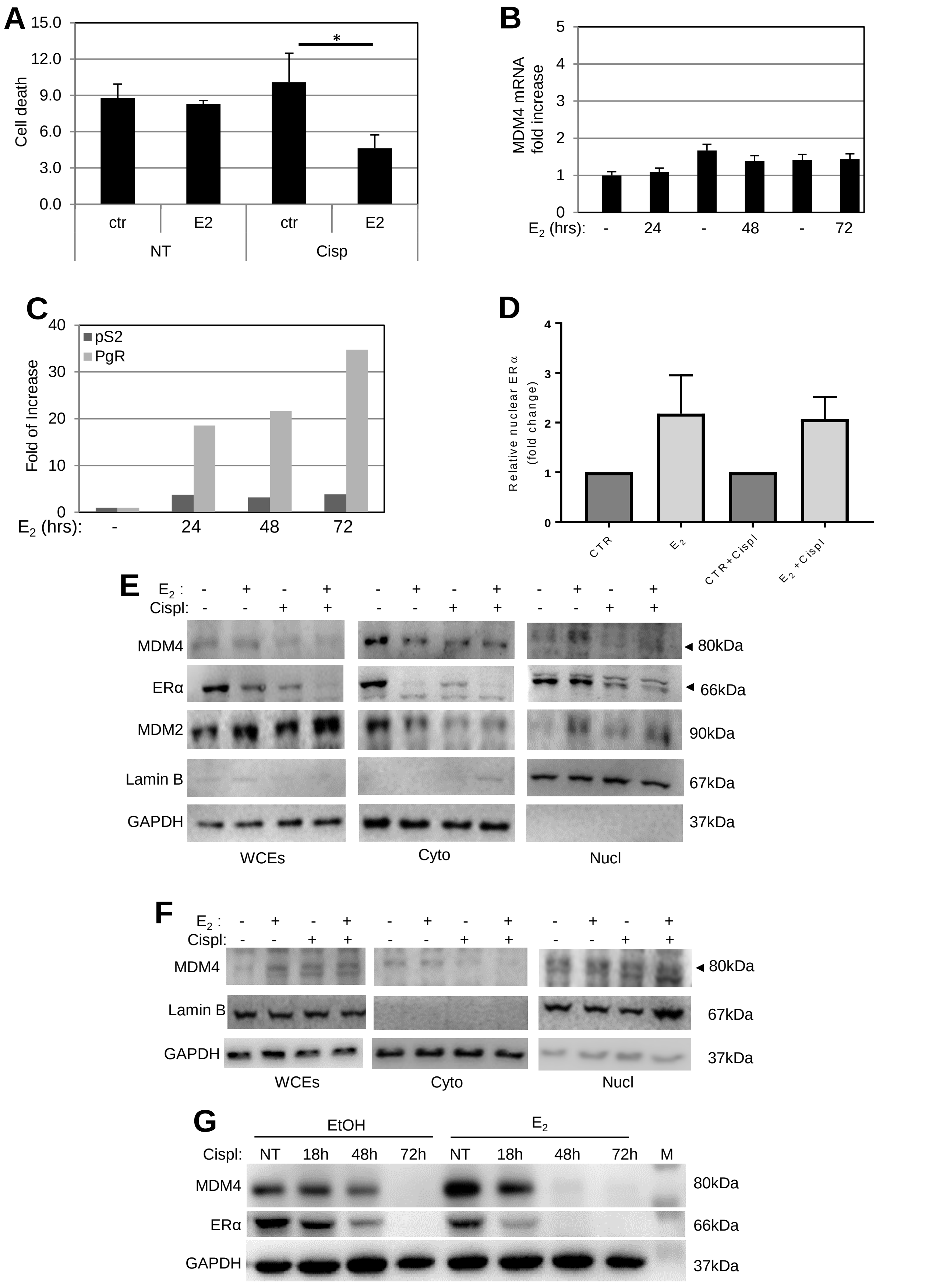

Figure S3. Analysis of estrogen pathway and MDM4 in estrogen-sensitive and –insensitive tumor cell lines. **A**, Cell death by adenylate kinase (AK) detection kit in T-47D cells. (*p=0.04, two-tailed unpaired t-test). **B, C,** qPCR of MDM4 (B) and PgR and pS2 (C) mRNA in MCF-7 cells treated as indicated. **D,** Nuclear levels of Erα relative to total levels in MCF7 cells treated as indicated. **E, F, G,** WB analysis of WCEs and subcellular fractions of T-47D (E), MDA-MB-231 (F) and MCF7 (G) cells treated as indicated.

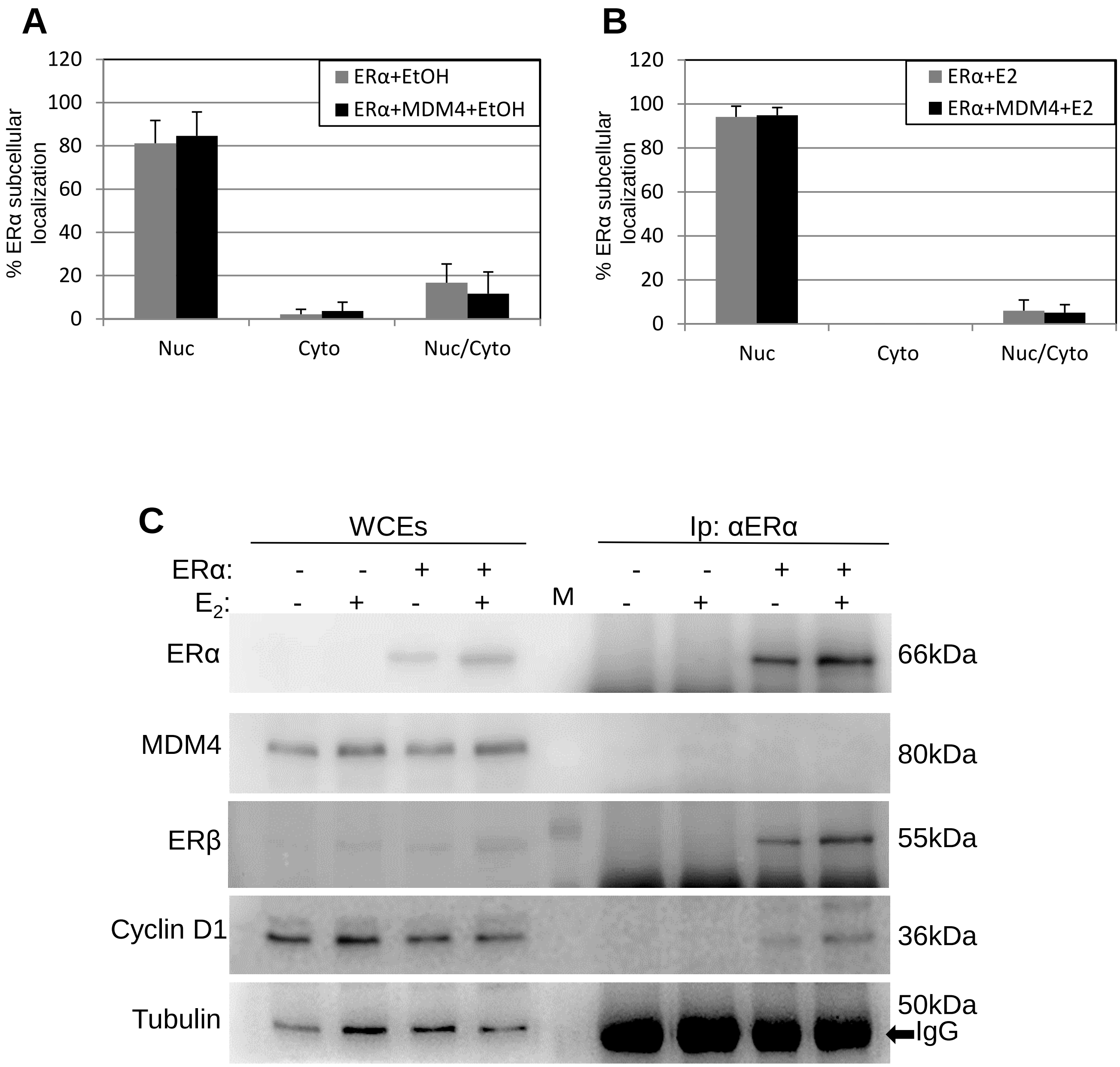

Figure S4. Analysis of ERα localization and ERα /MDM4 interaction. **A, B,** Graphs show percentage of A2780 cells showing only Nuclear (Nuc), or Cytoplasmic (Cyto) or Nuclear and Cytoplasmic (Nuc/Cyto) ERα signal relative to the total number of positive cells under indicated treatments. Mean±SD of 3 independent biological replicates is shown. **C,** Analysis of indicated proteins in coimmunoprecipitates of ERα exogenously expressed in A2780 cells: WCEs represent 1/50 of protein amount used for Ip. The data are representative of four biological replicates.

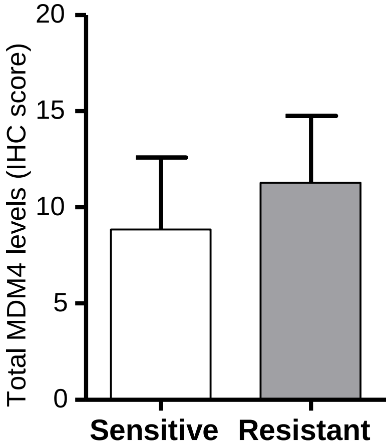

Figure S5. Analysis of total MDM4 levels in HGSOC patients. Graph shows total MDM4 levels (IHC immunoreactive score, mean±SD) in sensitive and resistant patients. Total MDM4 levels for each tumor were calculated as the sum of the nuclear and nuclear/cytoplasmic score (two-tailed unpaired t-test, $n=33$, $DF=31$, $t=1.784$, $p=0.084$).

Table S1. P53 status in fibrosarcoma of Mdm4^{Tg} and WT mice

N.	Mouse #	Genotype	Gender	P53 status	Mutation
1	360	Tg10	Female	WT	
2	365	Tg10	Female	WT	
3	367	Tg10	Female	MUT	R59L, G62R, Q67H, A80S, K152N
4	362	Tg10	Male	MUT	K159N
5	363	Tg10	Male	MUT	S34T
6	364	Tg10	Male	WT	
7	355	Tg15	Female	MUT	R246P
8	101	Tg15	Female	MUT	G241V
9	102	Tg15	Female	MUT	C235F
10	343	Tg15	Female	WT	
11	341	Tg15	Male	MUT	R155P
12	344	Tg15	Male	WT	
13	375	WT	Female	MUT	R339P
14	374	WT	Female	MUT	R246P
15	352	WT	Female	MUT	V170L
16	358	WT	Female	MUT	E343Stop
17	359	WT	Female	WT	
18	361	WT	Female	WT	
19	350	WT	Female	WT	
20	294	WT	Male	WT	
21	298	WT	Male	MUT	Q327H
22	370	WT	Male	WT	

Table S2. mRNA levels of indicated genes in thymocytes from WT and Mdm4^{Tg} mice

Gene			WT#	Mdm4^{Tg}	P value
Bax	Female	noIR	1,44±0,23	1,66±0,03	n.s.
		IR	11,2±2,3	13,16±6	n.s.
	Male	noIR	2,19±0,78	1,97±0,08	n.s.
		IR	13,55±2,1	12,81±2,5	n.s.
PMAIP1/Noxa	Female	noIR	0,2±0,1	0,2±0,06	n.s.
		IR	6,6±1,4	7,2±2,6	n.s.
	Male	noIR	0,33±0,23	0,22±0,05	n.s.
		IR	6,99±2,5	5,72±1,7	n.s.
BBC3/Puma	Female	noIR	0,21±0,2	0,13±0,004	n.s.
		IR	1,1±0,6	2,18±2	n.s.
	Male	noIR	0,19±0,08	0,17±0,009	n.s.
		IR	1,89±0,9	2,82±1,7	n.s.
p21/Waf1	Female	noIR	0,1±0,01	0,14±0,09	n.s.
		IR	4,43±2,3	5,92±3,3	n.s.
	Male	noIR	0,4±0,3	0,31±0,04	n.s.
		IR	5,34±1,3	5,21±2,1	n.s.
LGALS3	Female	noIR	0,5±0,4	0,68±0,3	n.s.
		IR	4,48±3,9	3,1±3,1	n.s.
	Male	noIR	1,06±0,6	1,02±0,4	n.s.
		IR	4,37±3,2	3,78±1,4	n.s.

Number of animals: WT Females noIR=3, Mdm4^{Tg} noIR=4, WT Females IR=6, Mdm4^{Tg} IR=6, WT Males noIR=3, Mdm4^{Tg} noIR=4, WT Males IR=8, Mdm4^{Tg} IR=5

Table S3. Clinical-pathological features of the overall series

Characteristics	No. of patients (%)
**All cases**	33
**Median Age, years (range)**	64 (51-81)
**Grade**	
G2	4 (12.1)
G3	29 (87.9)
**FIGO Stage**	
III	31 (93.9)
IV	2 (6.1)
**Residual tumor**	
0 mm	21 (63.6)
> 0 mm	12 (36.4)
**Primary chemotherapy**	
Platinum/paclitaxel	28 (84.8)
Platinum-based	3 (9.1)
Other	2 (6.1)
**Chemosensitivity**	
Sensitive	22 (66.7)
Resistant	11 (33.3)

Uncropped blots of Supplemental Figure 1A

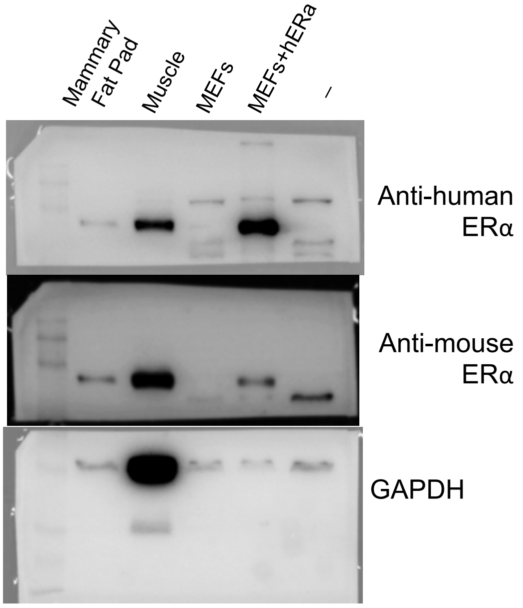

Uncropped blots of Supplemental Figure 3D

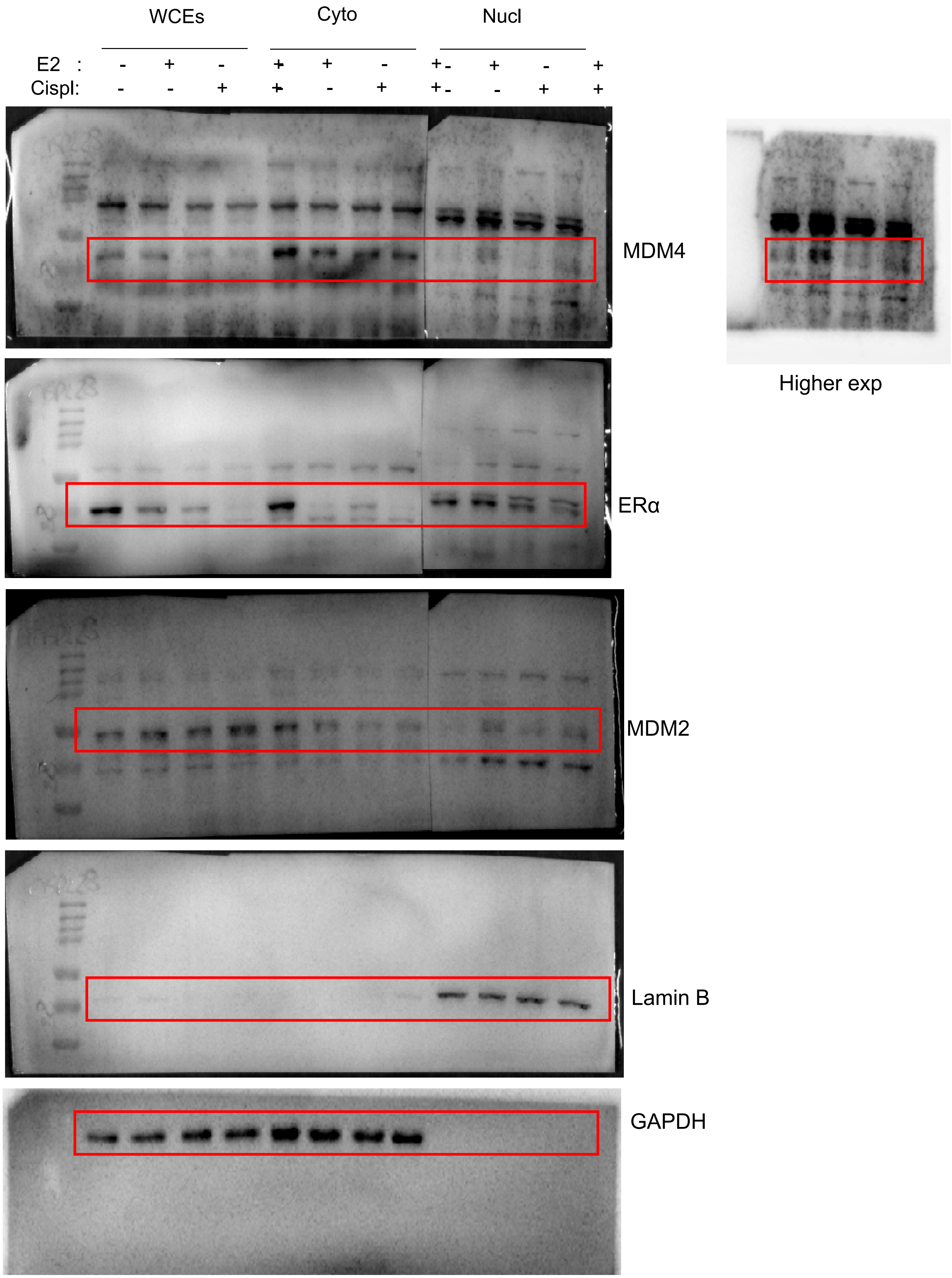

Uncropped blots of Supplemental Figure 3E

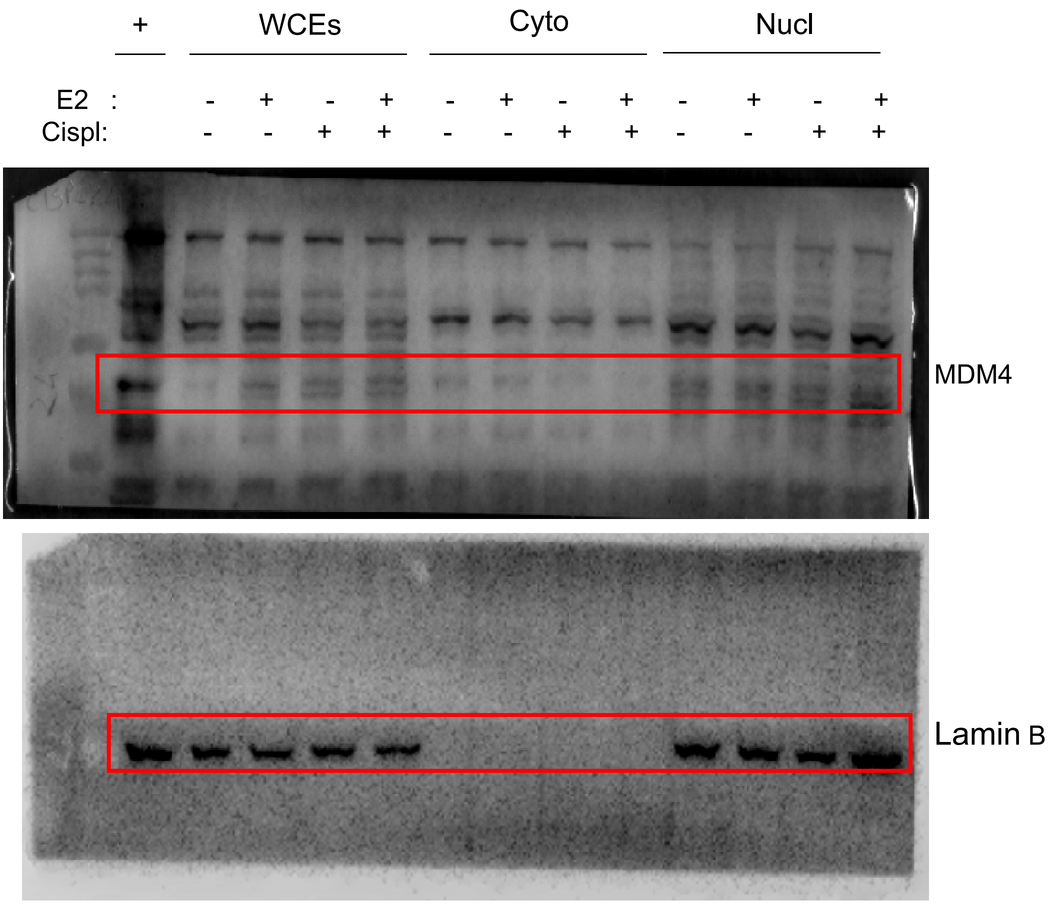

Uncropped blots of Supplemental Figure 4A

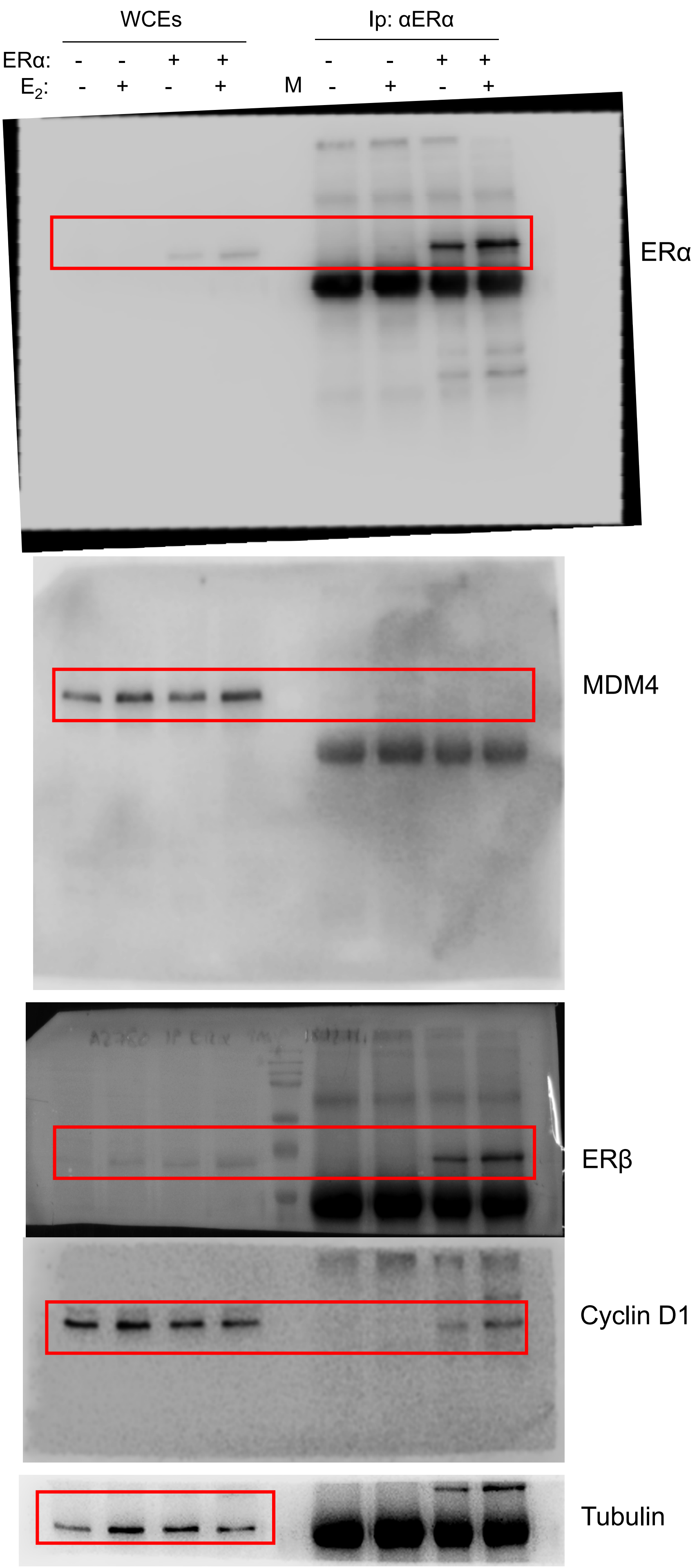

Fig 3c,d MDM4

Sample name	Volume	Height	Area
NT tot	27993,04	52,108	2,85
E2 tot	22816,26	41,735	2,85
NT CISPL tot	24714,32	41,603	2,565
E2 CISPL tot	19165,96	25,265	2,793
NT cito	23136,83	45,448	2,451
E2 cito	26099,4	50,217	2,793
NT CISPL cito	13947,01	28,381	2,508
E2 CISPL cito	14924,21	27,572	2,622
NT nucleo	4944,176	11,296	2,28
E2 nucleo	5525,907	12,257	2,451
NT CISPL nucleo	7259,189	16,543	2,28
E2 CISPL nucleo	10460,71	24,403	2,85

Fig 3c,d GAPDH

Sample name	Volume	Height	Area
NT tot	9711,844	21,063	2,544
E2 tot	8439,133	17,135	2,544
NT CISPL tot	7701,435	18,731	2,208
E2 CISPL tot	8526,761	18,254	2,544
NT cito	9255,905	22,829	2,064
E2 cito	11098,4	24,009	2,448
NT CISPL cito	7898,167	19,358	2,112
E2 CISPL cito	8545,655	18,44	2,544

Fig 3d LaminB

Sample name	Volume	Height	Area
NT nucleo	3440,243	11,526	2,064
E2 nucleo	3811,034	11,377	2,16
NT CISPL nucleo	3505,69	10,448	2,112
E2 CISPL nucleo	3639,619	9,617	2,4

Fig. 4a_MDM4

Number	Vol. %	Volume	Height	Area
No 1	19,979	15165347	38919	2378
No 2	9,274	7039599	19081	2030
No 3	11,781	8942458	20206	2378
No 4	21,029	15962413	29734	2668
No 5	23,187	17599912	31902	2320
No 6	14,75	11195840	19785	2494
No 7	9,553	7251087	11075	2262
No 8	11,267	8552637	9711	2668
No 9	8,237	6252524	9802	2494
No 10	10,049	7627508	12565	2146
No 11	9,746	7397378	11494	2320
No 12	8,976	6813443	6946	2262

Fig. 4a_GAPDH

Number	Vol. %	Volume	Height	Area
No 1	9,21	4142217	7358	2610
No 2	11,482	5164091	8671	2552
No 3	13,858	6232689	15326	1972
No 4	21,64	9732634	18231	2552
No 5	21,133	9504766	17827	2494
No 6	22,676	10198544	17226	2842
No 7	12,539	5639426	12821	2088
No 8	17,053	7669405	14112	2726
No 9	16,707	7514107	15390	2436
No 10	29,483	13259750	23617	2726
No 11	23,304	10481064	19285	2726
No 12	20,017	9002728	17186	2784

Fig. 4b_MDM4

Number	Vol. %	Volume	Height	Area
No 1	16,719	14970044	12217	2795
No 2	10,974	9825671	9505	2795
No 3	19,081	17085326	13257	3380
No 4	16,831	15070302	11127	2535
No 5	19,404	17374623	14465	2665
No 6	16,991	15213752	20521	2470
No 7	4,992	4469485	6432	2600
No 8	10,103	9046003	8864	2535
No 9	8,975	8035945	9488	2405
No 10	14,757	13213184	15328	2340
No 11	12,382	11087021	8850	3445
No 12	5,34	4781224	6581	3185

Fig. 4b_GAPDH

Number	Vol. %	Volume	Height	Area
No 1	11,562	3420026	5898	3185
No 2	10,396	3075056	3700	3185
No 3	12,021	3556013	4641	2990
No 4	10,306	3048568	4326	2730
No 5	19,026	5628062	10045	2665
No 6	36,689	10852748	15990	3120
No 7	5,327	1575815	3164	2795
No 8	15,702	4644646	7668	2795
No 9	11,059	3271415	5547	2665
No 10	20,095	5944285	9744	2730
No 11	21,977	6500885	10942	2665
No 12	14,051	4156478	6506	2990

Fig. 4c_MDM4

Number	Vol. %	Volume	Height	Area
No 1	7,222	2793825	5850	1944
No 2	14,243	5509721	8496	1998
No 3	19,933	7710931	11306	1944
No 4	19,167	7414764	12832	1836
No 5	20,149	7794582	12156	2106
No 6	19,286	7460561	10814	2268
No 7	25,331	9799232	13019	1998
No 8	45,775	17707641	17502	2268
No 9	51,284	19838839	24543	2160
No 10	44,135	17073363	18280	2052
No 11	46,801	18104763	17632	2160

Fig. 4c_LaminB

Number	Vol. %	Volume	Height	Area
No 1	24,474	26132054	59303	2320
No 2	20,63	22026834	59303	2494
No 3	11,326	12092594	43989	2668
No 4	18,197	19429105	60515	2552
No 5	9,636	10288722	32780	2552
No 6	15,738	16803499	52061	2262
No 7	8,101	8649621	31481	2668
No 8	8,814	9410679	29584	2552
No 9	6,345	6774201	28064	2378
No 10	16,706	17837419	58263	2552
No 11	23,629	25229003	60074	2726
